# Consumption of alcohol and blood pressure: Results of the ELSA-Brasil study

**DOI:** 10.1371/journal.pone.0190239

**Published:** 2018-01-08

**Authors:** Nathália Miguel Teixeira Santana, José Geraldo Mill, Gustavo Velasquez-Melendez, Alexandra Dias Moreira, Sandhi Maria Barreto, Maria Carmen Viana, Maria del Carmen Bisi Molina

**Affiliations:** 1 Postgraduate Program in Collective Health, Federal University of Espírito Santo (UFES), Vitória, Espírito Santo, Brazil; 2 Department of Maternal and child Nursing, Federal University of Minas Gerais (UFMG), Belo Horizonte, Minas Gerais, Brazil; 3 Graduate Studies Program of Public Health, Federal University of Minas Gerais (UFMG), Belo Horizonte, Minas Gerais, Brazil; Medical University Innsbruck, AUSTRIA

## Abstract

**Background:**

Prevention and reduction of excessive use of alcohol represents damages to society in general. In turn, arterial hypertension is the main attributable risk factor premature life lost years and disability.

**Objective:**

To investigate the relationship between alcohol consumption and high blood pressure in participants of the Brazilian Longitudinal Study of Adult Health (ELSA-Brasil).

**Methodology:**

A baseline data of total of 7,655 participants volunteers between 35 and 74 years of age, of both genders, in six educational and research institutions of three different regions of the country were interviewed between 2008–2010. Socioeconomic, haemodynamic, anthropometric and health data were collected in the research centers of ELSA-Brasil. The presence of high blood pressure was identified when the systolic blood pressure was ≥140 mm Hg and/or the diastolic was ≥90 mm Hg. Alcohol consumption was estimated and categorized regarding consumption and pattern of ingestion. The Student’s t-test, chi-squared and logistic regression tests were used for analysis, including potential co-variables of the model, and a 5% significance level was adopted.

**Results:**

A dose-response relation was observed for the consumption of alcohol (g/week) in systolic blood pressure and diastolic blood pressure. Alcohol consumption was associated with high blood pressure in men who reported moderate (*OR* = 1.69; _95%_CI 1.35–2.11) and excessive (*OR* = 2.70; _95%_CI 2.04–3.59) consumption. Women have nearly three times more chance of presenting elevated blood pressure when presenting excessive consumption (*OR* = 2.86, _95%_CI 1.77–4.63), and binge drinkers who drink more than 2 to 3 times a month have approximately 70% more chance of presenting with elevated blood pressure, after adjusting for consumption of drinks with meals.

**Conclusion:**

The consumption of alcohol beverages increases the odds of elevated blood pressure, especially among excessive drinkers. Therefore alcohol consumption needs a more robust regulation in view of its impact on population health.

## Introduction

Elevated blood pressure (BP) is a major risk factor for cardiovascular disease (CVD), causing significant loss of years of quality of life [1,2]. In addition, hypertension is a multifaceted disease, asymptomatic and difficult to control [3]. In Brazil, the prevalence of hypertension is high [4–7] giving a significant contribution to the overall cardiovascular risk. Some risk factors, such as smoking, have steadily decreased in recent decades [8] while others have increased such as obesity [9] and alcohol consumption [10]. In addition regular alcohol consumption is occurring increasingly earlier in the life [11], thus also providing an increase in general morbidity and mortality [12].

The relationship between alcohol consumption and BP is still controversial. With respect to the average alcohol consumption compared to non-drinkers throughout life, a J-shaped association is observed [13]. However, a cohort study, in 8,334 North Americans (45 to 64 years at baseline) showed a linear relationship between alcohol intake and BP, even at lower quantities after a six year follow up [14]. Another study in 50 centers worldwide in 9,681 men and woman, aged 20 to 59 years, showed positive association only with higher intake [15]. The consumption pattern of binge drinkers is also associated with higher BP levels compared to non-consumers [16]. These effects on BP can also be observed in the short term [17,18] and seem to differ between the sexes, being more likely to be raised by alcohol consumption in male drinkers [19,20].

Not only the frequency and number of doses per occasion, but also if consumption is with meals, may have an influence on the odds of developing hypertension The alcohol intake outside meals increases the likelihood of hypertension and is associated with BP increase in normotensive subjects [21,22]. Thus, reducing alcohol intake, by decreasing the amount of alcohol drunk or by choosing drinks with lower alcohol concentrations, consistently reduces BP levels [17,18].

Although other studies have already confirmed the harmful effects of alcohol consumption on the cardiovascular system, especially by elevating BP levels, the strength of this relationship was not investigated in the Brazilian population.

Considering the multifactorial nature of the BP elevation and hypertension development, our aim was to estimate the alcohol consumption of participants from the baseline of the Brazilian Longitudinal Study of Adult Health (ELSA-Brasil), as well as to analyse the relationship between alcohol consumption and BP.

## Materials and methods

This is a cross-sectional study conducted using the baseline data of the ELSA-Brasil study. The main objective of this study is to investigate the incidence of and risk factors for chronic diseases in the Brazilian population, in particular cardiovascular diseases and diabetes. Baseline data were collected 2008 to 2010 in 15,105 active and retired civil servants of both sexes, aged 35–74 years, from five universities and one research institution [23].

Subjects were identified for scheduling a visit to the research center to conduct the examinations and application of questionnaires (detailed description can be found in a previous report [24]). All research centers have received training in order to maintain data quality and safety [25]. Sociodemographic, health status and lifestyle data, including alcohol consumption, were collected during an interview. The self-reported race/skin colour was categorized as whites and non-whites (blacks, *pardos*, yellows and natives); education level was categorized as years in school as primary (≤ 8 y), secondary (9–11 y) and higher (≥12 y); smoking was divided in never, ex-smoker and current smoker; presence or absence of family history of hypertension and history of natural menopause were considered as dichotomous variables. Physical activity was estimated from the International Physical Activity Questionnaire (IPAQ) long version, in the domains of leisure time physical activity (LTPA) and displacement physical activity (DPA). The instrument consists of questions concerning the frequency, duration and intensity (LTPA: weak, moderate and vigorous; DPA: walking, cycling) physical activities [26]. The physical activity pattern, in its different domains, was recorded in minutes/week, consisting of multiplying the weekly frequency by the duration of each activity. Subjects were considered as physical activity when performing at least 10 minutes per week. The variable was later categorized as weak (sedentary), moderate and strong.

The anthropometric variables (weight, height and waist circumference) were measured with participants in fast, by trained assistants according to standard protocols. An electronic balance was used, with a capacity of 200 kg and a precision of 50g. The height was measured on a wall stadiometer with 1 mm precision, with an individual in a supine position, barefoot, leaning on the head, buttocks and heels on the wall and stare horizontally. The stature was verified in the inspiratory period of the respiratory cycle. The waist circumference (WC) was measured with the participant in an upright position breathing normally, with the feet together, the upper part of the dress erected and the arms crossed in front of the chest. The measurement was performed with an inextensible tape measure at the midpoint between the iliac crest and the lower edge of the costal arch [24,25].

The Body Mass Index (BMI) was calculated and cohort points were used to classify the nutritional status. Overweight was diagnosed when BMI ≥25 kg/m^2^. The WC and World Health Organization (WHO) criteria were used to identify abdominal obesity. The diagnosis of abdominal obesity was established from the following WC values ≥80 and ≥94 cm for women and men, respectively [27].

BP was measured after five minutes of rest using an oscillometric device (Omron HEM 705CPINT). Three measurements were taken at intervals of one minute, and the average of the last two considered casual BP [25]. BP levels were considered high in presence of systolic BP (SBP) ≥140 mm Hg and/or diastolic BP (DBP) ≥90 mm Hg [28].

Information about the use of medications to control hypertension was obtained from the interview by answering the question: ‘Have any of the medications you took in the last 2 weeks been for hypertension (elevated BP)?’ The classes of anti-hypertensive drugs were diuretics, β-blockers, ACE inhibitors, angiotensin receptor blockers, calcium channel blockers, direct vasodilators, central acting agents, mineralocorticoid receptor antagonists and renin inhibitors.

To evaluate the consumption of alcoholic beverages (beer, wine, spirits—rum, whiskey, ‘cachaça’ and vodka), we used the Alcohol Use Questionnaire (AUQ), which was structured with closed questions based on the questionnaire of the National Center for Health *Statistics*. The drinking pattern and weekly frequency of alcohol consumption in beverages was determined with this instrument [29].

For the calculation of the amount of ethanol in grams, the average alcoholic percentage of the most common beverage brands on the market was used: beer = 6%; wine = 12%; spirits = 39%. First, the amount reported weekly by the equivalent measurement in mL was determined. Then, the amount of pure alcohol intake in mL/week was calculated according to the alcoholic concentration of each beverage. Subsequently they were added to the amount of alcohol consumed and kinds of beverage and lastly multiplied by the density of ethanol (0.8) in order to obtain the total amount of pure ethanol in g/week.

Excessive drinkers were categorized as those with an ethanol consumption ≥210 and ≥140 g/week, for men and women, respectively [30]. Binge drinking was defined as consuming 5 or more drinks within a two hour period more than once a month [31]. The frequency alcoholic beverages consumption at meals was evaluated by the question ‘Considering all the alcoholic beverages that you consume, how often do you drink with meals?’ with the following possible answers: “Most frequently with meals”, “Both with and outside of meals” and “Most frequently outside of meals”.

**Fig 1:**
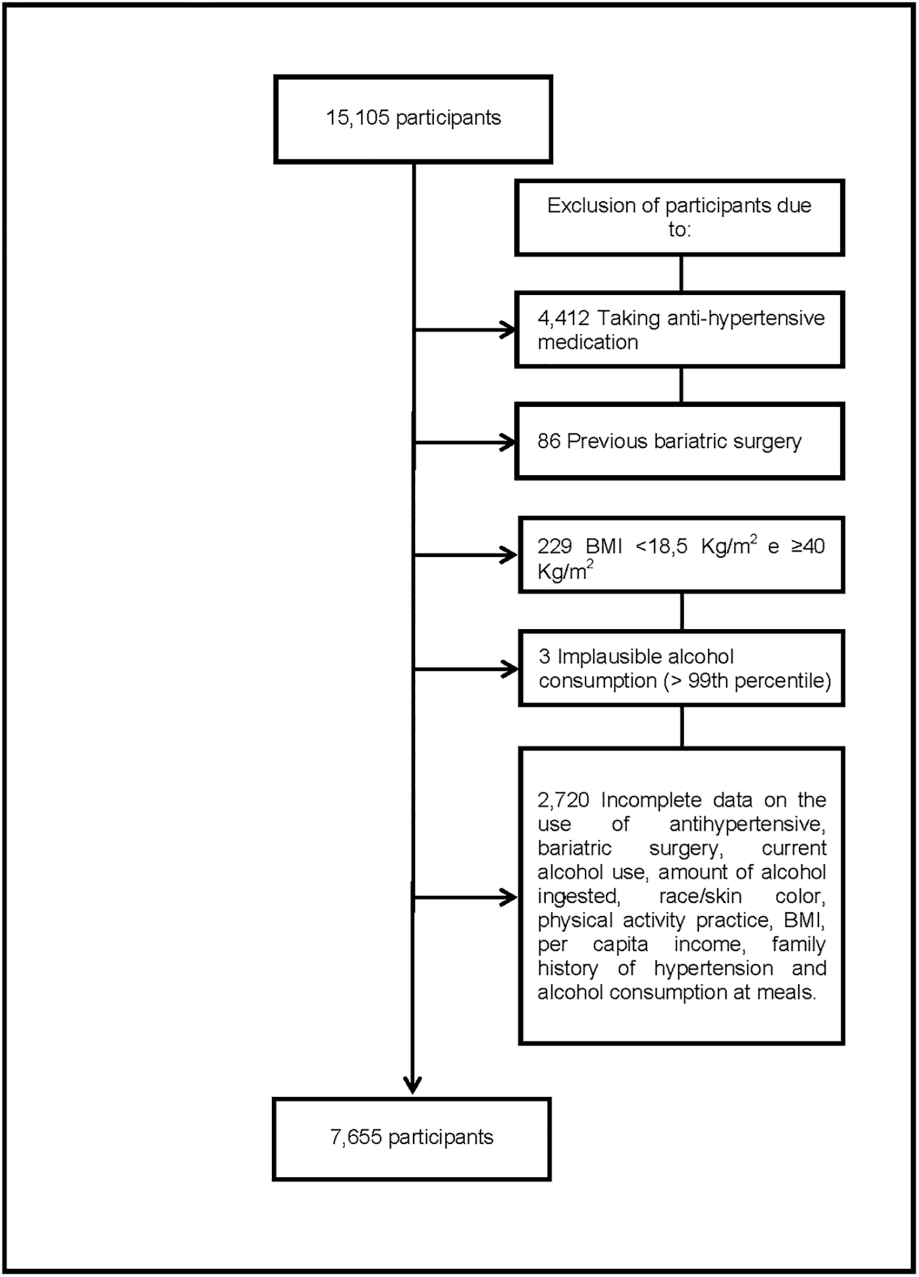
Participants exclusion flowchart.

We excluded from the present analysis, the participants reporting use of any anti-hypertensive drug class, subjects with previous bariatric surgery, those with a BMI <18,5 kg/m^2^ and ≥40kg/m^2^ and those with a calculated implausible alcohol consumption (above 99^th^ percentile) as well as participants with incomplete data related to alcohol consumption, race/skin colour, physical activity, per capita income or family history of hypertension (n = 7,450) resulting in a final sample of 7,655 individuals (Fig 1). We opted to exclude participants who reported using antihypertensive medication, since these drugs reduce BP levels, thus affecting the main outcome studied of interest.

The ELSA-Brasil was approved by the Research Ethics Committee of all participating institutions (Federal University of Rio Grande do Sul; University of São Paulo, Oswaldo Cruz Foundation, Federal University of Minas Gerais, Federal University of Espírito Santo and Federal University of Bahia) and all participants signed the Informed Consent Form.

Data were processed and analyzed using Stata statistical *software*, version 12.0. Values were expressed as mean and standard deviation (SD) or percentage. The variables were compared among the participants according to the BP classification using Student’s T and chi-square tests. We conducted logistic regression models including the potential confounders. In the modeling process, the exposure variables were included from the knowledge of their potential association with the outcome variable, considering a level of significance of 20% (*p* ≤ 0.20). Thus, all variables with p ≤ 0.20 were introduced into the model and, later, those that did not remain statistically significant (*p*> 0.05) were removed. When not adjusted, the gender was analyzed separately due the difference in alcohol intake. Statistical significance was set at 5%.

## Results

The final sample consisted of 7,655 individuals (50.1% men) with an age mean of 50 ± 9 years. Of these, 52.5% were white, 53% had higher education and the average per capita income was R$ 1701.00 (equivalent to US$ 1028.00) at the time of baseline. Overweight was present in the majority of participants (57.6%), as well as abdominal obesity (55.6%). 56.8% were never smoked, 75.7% related to perform weak physical activity (sedentary) and 68.3% reported a family history of hypertension (68.3%). Participants with a family history of hypertension had a lower mean age (50 ± 8 years) than those who did not (52 ± 9 years).

Approximately 12% of the sample included in this analysis showed elevated BP level, this being two times more prevalent in men (16.6%) than in women (7.5%), seven times more prevalent among current alcohol users (71.5%) and almost twice as much among former users (18.4%), compared with those who reported never having consumed alcohol in their lives (10.1%). The number of abstainers among women was almost four times higher than in men. Among former alcohol users (n = 1,855), 73% reported having discontinued use more than two years ago, 29% reported health problems and 13% discontinued because of medical advice. Among those who reported current alcohol consumption, 14.1% were classified as excessive drinkers and 25.3% as binge drinking. Beer was the most reported type of drink (78.8%) with an average consumption of 1,906 mL/week and more than half (53.8%) used alcohol most frequently outside of meals.

Table 1 shows characteristics of the participants, according to BP classification (normotensive and high BP). All the variables presented significant differences, except for the presence of family history of hypertension. The normotensive individuals presented higher mean per capita income, and more frequent alcohol intake with meals, schooling and practice of strong physical activity. The group with elevated BP, was older, and showed higher body weight, BMI and WC. Regarding behavioral characteristics, this group also showed higher alcohol consumption (g/week) and higher percentage of alcohol users, smokers and excessive and binge drinking. Most were male (68.8%) and non-whites (61.9%).

**Table 1:** Characteristics of participants, by blood pressure classification. ELSA-Brasil, 2008–2010 (n = 7,655).

Variables	Normotensive (n = 6,732)	Elevated blood pressure (n = 923)	Value of p*
	Mean±SD	Mean±SD	
Age (in years)	50±8	53±9	<0,001
Weight (kg)	71.7±13.3	76.7±14.9	<0,001
Income per capita (R$)	1745±1417	1384±1248	<0,001
Body Mass Index (kg/m^2^)	26±3.9	27.4±4.2	<0,001
Waist Circumference (cm)	88.6±11.1	94.4±11.5	<0,001
Alcohol consumption (g/week)	100±108	149±148	<0,001
Family history of hypertension (%)	68.2	69.3	0,487
Current alcohol users (%)	62.3	71.5	<0,001
Smoking (%)	14.5	16.8	0,004
Excessive drinkers (%)^a^	12.5	24.6	<0,001
Binge drinking (%)^a^	23.6	36.1	<0,001
More frequent alcohol consumption with meals (%)^a^	40.1	29.6	<0,001
Higher education (%)	55.2	36.9	<0,001
Strong physical activity practice (%)	10.5	6.5	<0,001

^a^ n = 4,857

R$: Brazilian reais; 2009 conversion rate of 1.8 Brazilian reais = 1 US dollar.

*p-value from Student’s T and chi-square tests.

**Fig 2:**
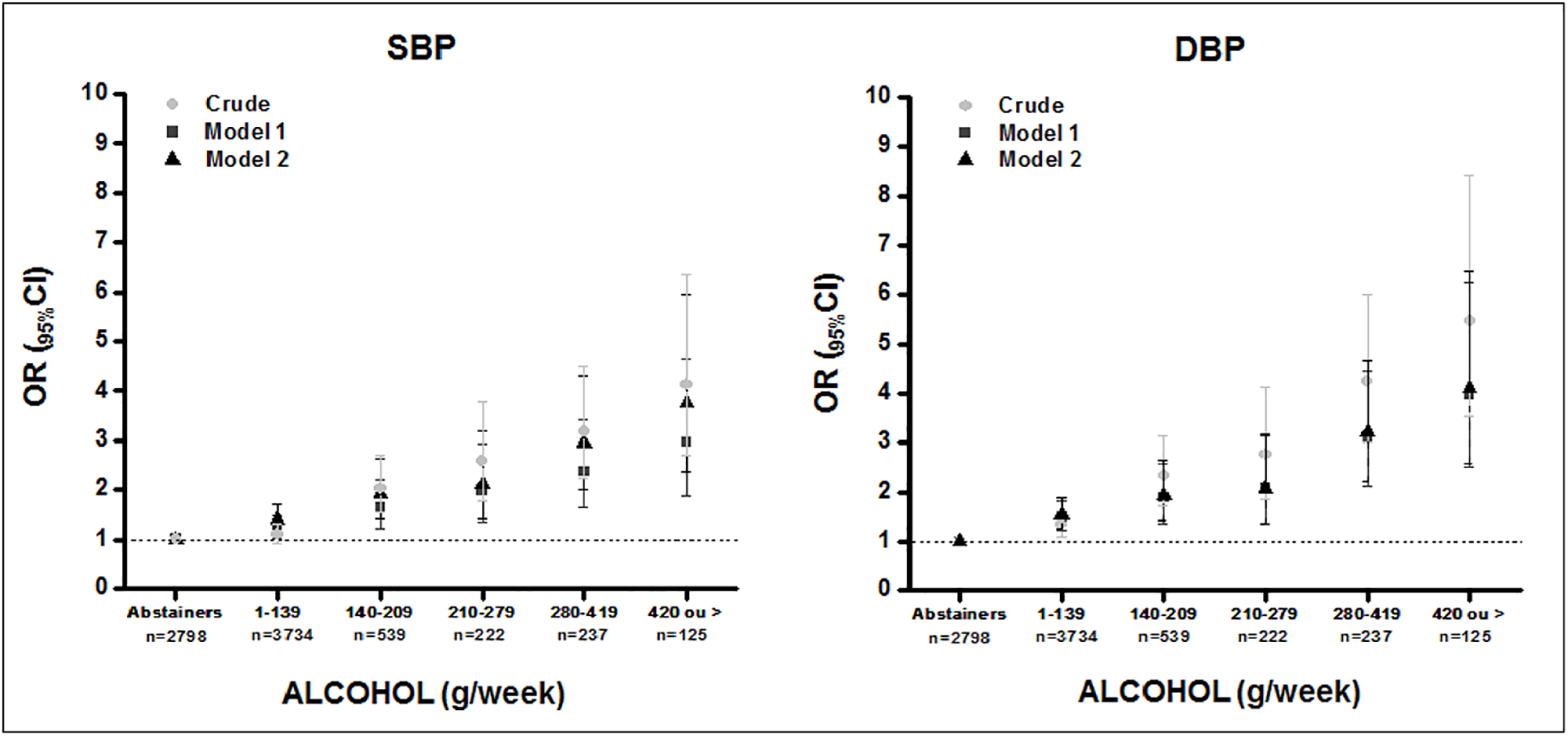
Association between the categories of alcohol consumption (g/week) and elevated systolic and diastolic blood pressure. ELSA-Brasil, 2008–2010 (n = 7,655). Model 1: Adjusted for sex, race/skin colour, income per capita, physical activity, smoking, abdominal obesity. Model 2: model 1 + adjusted for age, family history of hypertension.

The crude and adjusted associations of elevated SBP and DBP with the categories of alcohol consumption, divided into the amounts of ≥1 to <140; ≥140 to <210; ≥210 to <280; ≥280 to <420; and ≥420 g/week, are presented in Fig 2. Alcohol consumption, when compared to abstention, was associated with elevated SBP in all categories of consumption even after adjusting (in model 1) for sex, race/ skin colour, income per capita, physical activity, smoking and abdominal obesity. There was a progressively increased odds of elevated SBP as the alcohol consumption (g/week) increased (≥1 to <140, *OR* = 1.25, _95%_CI 1.01–1.49; ≥140 to <210, *OR* = 1.63, _95%_CI 1.21–2.20; ≥210 to <280, *OR* = 1.98, _95%_CI 1.43–3.94; ≥280 to <420, *OR* = 2.37, _95%_CI 1.6–3.42; ≥420, *OR* = 2.95, _95%_CI 1.88–4.64).

In Fig 2, model 2, adjusted for all variables of the first model with the addition of age and family history of hypertension, it was observed that alcohol consumption (g/week) remained associated with elevated SBP, according to the alcohol consumption category (≥1 to <140, *OR* = 1.41, _95%_ CI 1.15–1.71; ≥140 to <210, *OR* = 1.93, _95%_ CI 1.42–2.63; ≥210 to <280, *OR* = 2.11, _95%_ CI 1.41–3.18; ≥280 to <420, *OR* = 2.95, _95%_ CI 2.02–4.31; ≥420, *OR* = 3.75, _95%_ CI 2.37–5.96).

Taking as reference the abstention group, alcohol consumption also was associated with elevated DBP in all categories of consumption, demonstrating a showing effect (≥1 to <140, *OR* = 1.49, _95%_CI 1.21–1.83; ≥140 to <210, *OR* = 1.87, _95%_CI 1.37–2.57; ≥210 to <280, *OR* = 2.07, _95%_ CI 1.36–3.15; ≥280 to <420, *OR* = 3.08, _95%_ CI 2.13–4.47; ≥420, *OR* = 3.96 IC _95%_, 2.51–6.25). This relationship was still present in the full adjusted model 2 (≥1 to <140, *OR* = 1.54, _95%_ CI 1.26–1.90; ≥140 to <210, *OR* = 1.93, _95%_ CI 1.41–2.64; ≥210 to <280, *OR* = 2.07, _95%_ CI 1.36–3.16; ≥280 to <420, *OR* = 3.21, _95%_ CI 2.21–4.66; ≥420, *OR* = 4.10, _95%_ CI 2.59–6.47) (Fig 2).

Although there was no interaction, there was a significant difference in alcohol consumption between men (136 ± 133 g/week) and women (66 ± 70 g/week) (*p* <0.001). Table 2 shows the gross and adjusted association of high BP with alcohol consumption categories (abstemious, moderate and excessive) by sex. After adjustment to all potential confounders (model 2), moderate (≥1 to <210 g/alcohol per week) and excessive (≥210 g/alcohol per week) consumption were associated with greater chances of having elevated BP (*OR* = 1.69 _95%_CI 1.35–2.11; *OR* = 2.70 _95%_CI 2.04–3.59) in men compared to the abstainers. In contrast, only excessive drinking (≥140 g/alcohol per week) showed significant association in women (OR = 2.86, _95%_ CI 1.77–4.63).

**Table 2:** Odds ratio (OR) for elevated systemic blood pressure according to alcohol consumption. ELSA-Brasil, 2008–2010 (n = 7,655).

	*n*	Crude Model		Model 1		Model 2		Model 3	
		*OR*	(_95%_CI)	*OR*	(_95%_CI)	*OR*	(_95%_CI)	*OR*	(_95%_CI)
**Male**									
Abstemious	**1054**	**1**		**1**		**1**			
Moderate	**2273**	1.30	(1.05–1.61)	1.55	(1.25–1.94)	1.69	(1.35–2.11)	**1**	
Excessive	**507**	2.50	(1.91–3.26)	2.48	(1.87–3.27)	2.70	(2.04–3.59)	1.52	(1.19–1.94)
**Female**									
Abstemious	**1744**	**1**		**1**		**1**			
Moderate	**1898**	0.96	(0.75–1.23)	1.16	(0.89–1.51)	1.28	(0.98–1.67)	**1**	
Excessive	**179**	2.15	(1.36–3.38)	2.42	(1.51–3.88)	2.86	(1.77–4.63)	2.04	(1.27–3.27)
**ELSA-Brasil**									
Abstemious	**2798**	**1**		**1**		**1**			
Moderate	**4171**	1.31	(1.12–1.53)	1.36	(1.15–1,61)	1.50	(1.26–1.77)	**1**	
Excessive	**686**	2.98	(2.40–3.70)	2.36	(1.87–2,99)	2.64	(2.08–3.35)	1.63	(1.31–2.02)

Model 1: Adjust for race/skin colour, income per capita, physical activity, smoking, abdominal obesity and sex when analyzed throughout the ELSA-Brasil population.

Model 2: model 1 + adjust for age, family history of hypertension and menopause.

Model 3: model 2 + adjust for consumption of alcohol more often with meals.

Abstemious: 0g/ethanol/week (male and female).

Moderate: 1 to 209g/ethanol/week (male); 1 to 139g/ethanol/week (female).

Excessive: ≥210g/ethanol/week (male); ≥140g/ethanol/week (female).

When added adjusted for drinking alcohol most frequently with meals (model 3), and changing the reference group to moderate consumers, there was an association between excessive drinkers with an increased chance of having elevated BP both in men (*OR* = 1.52, _95%_ CI 1.19–1.94), women (*OR* = 2.04, _95%_ CI 1.27–3.27) and ELSA-Brasil population (*OR* = 1.634, _95%_ CI 1.31–2.02).

Table 3 shows the adjusted OR for elevated BP, for different categories of binge drinkers, according to frequency of use. In the first series of logistic regression analyses, drinkers those who never reported binge drinking (five or more drinks in a two-hour) were used as reference. There was a significant increase in BP levels in individuals who reporting this behaviour from 2 to 3 times per month. Women, whites, physically active subjects, and most frequently alcohol consumption with meals were inversely associated with BP elevation (Table 3).

**Table 3:** Odds ratio (OR) for high systemic blood pressure according to the pattern of alcohol consumption. ELSA-Brasil, 2008–2010 (n = 4,857).

Binge Drinking	*n*	Crude Model		Model 1		Model 2	
		*OR*	(_95%_CI)	*OR*	(_95%_CI)	*OR*	(_95%_CI)
Never	**1617**	**1**		**1**		**1**	
Occasionally	**2012**	1.33	(1.08–1.64)	1.18	(0.95–1.47)	1.17	(0.94–1.46)
2-3x/month	**415**	2.05	(1.52–2.75)	1.69	(1.23–2.32)	1.66	(1.20–2.28)
1-2x/week	**722**	2.1	(1.64–2.69)	1.49	(1.14–1.94)	1.43	(1.09–1.87)
Almost daily or >1x/day	**91**	3.22	(1.96–5.27)	2.15	(1.27–3.64)	2.05	(1.21–3.47)

Model 1: Adjusted for Sex, age, race/skin colour, per capita income, physical activity, smoking, abdominal obesity, family history of hypertension.

Model 2: model 1 + adjusted for consumption of alcohol more often with meals.

For comparison purposes, participants who had received medical information that at some point in their life they had hypertension were excluded. The results found were similar to those found in the present study, with a small reduction in power due to the sample size, mainly in the most frequent consumption categories.

## Discussion

The findings of this study confirm that alcohol consumption was associated with an increase in the odds of elevated BP in in both sexes. No protective effect of alcoholic beverages intake was found even in moderate amounts. Additionally there was a linear trend in this association. Increase binge drink pattern frequency did not show increase excess of odds.

The positive linear trend relationship found in this study is not in agreement with other studies [13]. There was no evidence of a protective effect of alcohol against BP increase low levels of alcohol consumption. Sesso et al. [19] also reported in a longitudinal study in workers a gender difference on the effects of ethanol consumption on the risk of hypertension. An intake greater than or equal to two daily doses of alcoholic beverages, which translates into approximately 200 g/week, was related to an increased risk of this disease. However, even in their study, women displayed a possible J curve since low to moderate consumption was associated to a modest reduction in the risk of hypertension. On the other hand, in those with excessive consumption, equal to or greater than four doses per day, the relationship was different, increasing significantly.

It is worth noting that the SBP seems to be more responsive to modifiable risk factors such as to moderate or strong alcohol intake [8]. This association is of concerning because the SBP exerts higher influence on cardiovascular risk than DBP [32,33] and thus becomes important, since it demonstrates the need for other possible approaches to reduce BP levels.

The results showed that men consume drinks more often and are more excessive drinkers than woman, indicating a sex-related difference on alcohol consumption patterns. Men are three times more frequent drinkers and intake about 80% more ethanol than women. These sex- differences tend to decrease among the younger [34].

Although there was no association between alcohol intake and BP in woman with moderate consumption, a greater difference was observed when the consumption was excessive. This findings differs from those by Pajak et al. [16] in a sample of adults, without use of anti-hypertensive medication, from Central and Eastern Europe, showed linear associations of high BP with annual ethanol intake, drinking frequency and binge drinking in women and no beneficial effect of moderate alcohol consumption.

There is agreement in the literature regarding the association of excessive consumption of alcohol with BP levels [14,16,20,32]. The frequency of binge drinking in this study (25,3%) was close to values estimated by WHO (22%) [7]. However, it was higher than the values found in the National Survey of Health 2013 (13.7%) and in a Brazilian survey obtained by phone calls (VIGITEL 2014;16.5%) [35,36]. This fact may be due to the differences between the survey methodologies.

The high perceptual of current drinkers, excessive drinkers and excessive episodic drinkers can be explained by the high level of education and average per capita income of the participants, since more than half of the sample reported having a higher education. Individuals with higher education have more opportunities to engage in excessive consumption than those with a medium level education, as well as those with higher incomes per capita compared with those with lower incomes [37].

Excess weight and abdominal obesity, present in more than half of the sample, were significantly higher among individuals with elevated BP. Luo et al. [38] in their findings reinforce the fact that abdominal obesity presents an interaction with alcohol consumption, increasing the risk of hypertension.

The alcohol consumption patterns of effects were also observed in a study of adult Brazilians living in six states from different regions of the country. A higher level of alcohol consumption, associated with more than seven drinks per week taken out of the meals, was most likely to lead to an elevation in BP [22].

The sensitivity analysis showed that the ELSA participants excluded from the present analysis (those taking anti-hypertensive medication, previous bariatric surgery or with an extreme BMI) differ from those included in our analyses in terms of gender, schooling, smoking, physical activity practice and age group. However, they are not different when analysed in terms in alcohol consumption (never, ex-consumer and current consumer).

The subjects taking anti-hypertensive medication (56±9 years) had a higher mean SBP and DBP, as well as greater alcohol consumption (g/week) and excessive alcohol consumption than those without medication (50±9 years). Despite the difference in the mean age subjects using medication was greater alcohol intake also reinforcing the need to change behaviour.

Ethanol consumption starting increasingly earlier in the Brazilian population is of concern as well as the increase in the quantities of alcoholic beverages ingested by occasion [10,11]. Early use of alcohol may be directly related to increased health and social problems arising from alcohol abuse in adulthood [39]. The implications of these findings are contrary to the desired advances in the control of alcohol consumption. The goal of reducing abusive alcohol consumption is compromised by this increase.

The limitations of the study include to the cross-sectional design and the non-assessment of possible biological mechanisms that may lead to increased BP levels in individuals more sensitive to alcohol consumption. Another important point is the non-assessment of alcohol consumption of abstainers in the past, which makes it difficult to know the profile of current abstainers. Finally, the classification of five or more doses in two hours and at least once a month for both sexes may have underestimated the number of episodic excessive drinkers, as it does not consider fewer doses for women since their consumption is lower than that of men.

The strength of the study is that the analyses were restricted to those who did not use anti-hypertensive medication in order to eliminate the possible effects of anti-hypertensive drugs on outcome, masking individuals with high BP. Complementary analysis with those with previous diagnosis of hypertension showed similar results. The exclusion of participants using medication eliminated the effects of reverse causality considering the negative relationship between hypertension treatment and alcohol intake. In addition, this is part of a large study with rigorous control on data collection and management [24,25,40].

## Conclusion

In conclusion, our study showed a positive relationship between alcohol consumption and odds of high BP in a large sample of adults. Excessive drinkers, habitual or binge, presented higher odds of high BP when compared to abstainers. The reduction of excessive alcohol consumption should be encouraged as well as other modifiable behaviour factors in the general population and especially in individuals at higher risk for hypertension development.

## Supporting information

S1 File Data available. (XLSX)

S2 File Captions for data available. (PDF)
