# Supplementary material for: Consumption of alcohol and blood pressure: Results of the ELSA-Brasil study
Source: PLoS One. 2018 Jan 8;13(1):e0190239. doi: 10.1371/journal.pone.0190239 (PMC5757983; doi:10.1371/journal.pone.0190239)
Supplement: S2 File — (PDF) [file pone.0190239.s002.pdf]

NAME	CAPTIONS
**ID**	Number of participants
**RCTA8**	Sex<br>Masculino = Men<br>Feminno = Woman
**IDADEA**	Age in years
**ANTA3**	Height (cm)
**HFDA07**	Family history of hypertension<br>Sim = Yes<br>Não = No
**A_PESO**	Weight (kg)
**A_IMC1**	BMI (kg/m2)
**A_CINT**	Waist circumference
**A_RENDAPERCAPITA**	Income per capita (Brazilian reais)
**A_FUMANTE**	Smoker<br>Nunca fumo = Never<br>Ex fumante = Ex-smoker<br>Fumante = Current smoker
**A_USODEALCOOL**	Alcohol use<br>Nunca usou = Never<br>Ex usuário = Ex- alcohol use<br>Usuário = Current alcohol use
**A_VOLALCGR**	Amount of alcohol ingested per week in g
**A_BEBEXCESSIVO**	Excessive drinker (M: ≥ 210 g/w; W: ≥140 g/w)<br>Sim = Yes<br>Não = No
**A_ATIVFISICA**	Physical activity at leisure<br>Fraca= Weak<br>Moderada= Moderate<br>Forte= Vigorous
**A_MEDANTHIPERT**	Use of antihypertensive medication<br>Sim = Yes<br>Não = No
**MEDIA_SIS**	Mean systolic blood pressure
**MEDIA_DIAS**	Mean diastolic blood pressure
**HA_SIS**	Presence of systolic blood pressure elevated<br>Sim = Yes<br>Não = No
**HA_DIAS**	Presence of diastolic blood pressure elevated<br>Sim = Yes<br>Não = No
**HA_SISTEMICA**	Presence of systolic and/or diastolic blood pressure elevated<br>Sim = Yes<br>Não = No
**CLASS_HAS**	Classification of blood pressure<br>Normal = Normal<br>Elevada = Elevated
**CLASS_EXCPESO_IMC**	Classification of the presence of excess weight according

	to BMI<br>Sim = Yes (BMI ≥ 25 kg/m^2)<br>Não = No (BMI < 25 kg/m^2)
**CLASS_EXCPESO_CC**	Classification of presence of excess weight according WC (M: ≥ 94cm; W: ≥80 cm)<br>Sim = Yes<br>Não = No
**CLASS_RACA_COR**	Race /colour classification<br>Branco = WhiteS<br>Não Branco = Non-whites
**CLASS_ESCOL**	Classification of schooling<br>Fundamental = Primary (≤ 8 y)<br>Médio = Secondary (9-11 y)<br>Superior = Higher (≥12 y)
**MENOPAUSA**	Natural Menopause<br>Sim = Yes<br>Não = No
**CONSUMODEALCOOL**	Current alcohol consumption g/week<br>Abstêmios = Abstemious (0 g/w)<br>Moderado = Moderate drinkers (M: 1 to < 210 g/w; W: 1 to <140 g/w)<br>Excessivo = Excessive drinkers (M: ≥ 210 g/w; W: ≥140 g/w)
**ALCOOLREFEICOES**	Consumption of alcohol with meals in 3 categories<br>Sempre ou maioria junto às refeições = Most frequently with meals<br>Tanto junto quanto fora das refeições = Both with and outside of meals<br>Nunca ou maioria fora das refeições = Most frequently outside of meals
**BINGE5CAT**	Frequency of binge drinking<br>Nunca = Never<br>Somente em ocasiões especiais = Occasionally<br>2 ou 3x por mês = 2-3x/month<br>1 a 2x por semana = 1-2x/week<br>Praticamente todos ou dias ou mais de 1x ao dia = Almost daily or >1x/day
**BINGE2CAT**	Binge drinking 2 categories (≥5 drinks within a two hour period more than once a month)<br>Sim = Yes<br>Não = No
**ALCOOLGSEMCAT**	Alcohol consumption g/week (categories)<br>Abstêmios = Abstemious<br>0 – 139,99 g/semana = 0 – 139,99 g/w<br>140 – 209,99 g/semana = 140 – 209,99 g/w<br>210 – 279,99 g/semana = 210 – 279,99 g/w<br>280 – 419,99 g/semana = 280 – 419,99 g/w<br>420 a 1074 g/semana = 420 a 1074 g/w
